# Supplementary material for: The fitness landscape of the African Salmonella Typhimurium ST313 strain D23580 reveals unique properties of the pBT1 plasmid
Source: PLoS Pathog. 2019 Sep 27;15(9):e1007948. doi: 10.1371/journal.ppat.1007948 (PMC6785131; doi:10.1371/journal.ppat.1007948)
Supplement: S1 Text — (PDF) [file ppat.1007948.s001.pdf]

# Supporting results

## <1% of coding genes show strong transposon orientation bias

To explore whether transposon orientation bias impacted upon the fitness data reported here, we searched for changes in orientation bias in the LB and InSPI2 output samples, with respect to the Input sample. We reasoned that, if a specific transposon orientation was strongly favoured in the output sample of the environmental conditions tested, and showed no orientation bias (or the opposite transposon orientation was favoured) in the input, then the favoured transposon orientation could have a negative or positive effect upon fitness compared with transposon insertions in the opposite orientation.

The transposon orientation score (column '*(sense - anti)/all*' in S8 Table) was used to define strong orientation bias in genes and intergenic regions when the amount of transposon insertions in one orientation was ≥3 times higher than the amount of transposons in the opposite orientation, corresponding to orientation scores of ≥0.5 or ≤-0.5. A Chi-squared test calculation indicated that, for a power of 0.8, the sample size (number of distinct insertion sites) needed to be at least 32. There were 3872 coding genes with ≥32 insertions in the Input sample. Among them, only 77 showed a significant orientation bias and only 15 of those showed a strong effect. Those results revealed that, for those coding genes with sufficient data available, only 0.4% showed strong orientation bias.

Specifically, 28 and 23 coding genes showed strong orientation bias in the LB and InSPI2 outputs, respectively. Of these, 12 coding genes in LB and 10 in InSPI2 also showed a significant orientation bias in the Input ($P \leq 0.05$), all in the same orientation as in the output samples. A total of 12 out of 561 intergenic regions with sufficient data available (1.8%) showed strong orientation bias in the Input sample. We identified 12 and 17 intergenic regions with strong orientation bias in the LB and InSPI2 outputs, respectively. Of these, 10 intergenic regions in LB and 11 in InSPI2 also showed significant orientation bias in the Input sample, in the same favoured orientation as in the outputs. For the macrophage samples, no coding genes or intergenic regions showed a strong orientation bias in the two output samples.

Overall, for those coding genes and intergenic regions with sufficient data available, >99% and about 98%, respectively, showed no evidence of a strong transposon orientation bias on the fitness defects reported in our study.

## Three passages in murine macrophages select for *S*. Typhimurium rough mutants

To determine whether the amount of bacterial cells of the D23580 transposon library used to infect macrophages contained a good representation of each individual mutant, a D23580 spontaneous mutant resistant to nalidixic acid (Nal) was added in a proportion 1:180,575 to the pool of kanamycin resistant mutants before infections. The D23580 transposon library was passaged three times in RAW264.7 macrophages, recovering intracellular bacteria at 8 h post-infection (p.i), and growing them in LB at 37ºC for 12 h, 220 rpm, between macrophage passages (S3A Fig). Three biological replicates

were performed, each one containing a pool of the intracellular bacteria from 12 wells from 6-well plates (Sarstedt). Ideally, the M.O.I. for screening mutants would be 1:1 to assume that one specific mutant infects one macrophage. However, due to the high complexity of the transposon library, an M.O.I. close to 10:1 was intended instead. The Nal resistant mutant initially mixed with the D23580 transposon pool was recovered after the second and third passages with a ratio of 1:300,000 and 1:381,250, respectively. We concluded that a good representation of mutants that do not exhibit fitness defects in our infection macrophage model would be seen across the three passages. The fold-change replication of bacteria in the intra-macrophage environment was calculated in each passage by comparing bacterial counts at 8 h p.i. versus 1.5 h p.i. The intra-macrophage replication of bacteria increased in the second passage compared to the first passage. Intriguingly, in the third passage bacteria exhibited less intra-macrophage proliferation than in the second and first passages.

To select for mutants with an increased fold-change replication inside macrophages in the third passage of macrophages, we hypothesised that extending the amount of time inside macrophages would improve selection. Therefore, we passaged the D23580 transposon library three times in murine RAW264.7 macrophages, collecting the intracellular bacteria at 12 h p.i. (S3B Fig). Results were similar to the previous passages collected at 8 h p.i., with an increased fold-change replication in the second passage versus the first passage. The fold-change replication values in the three passages were on average 5 times higher, comparing 12 h p.i. with 8 h p.i. The intra-macrophage replication of bacteria in the third passage was lower than in the second and the first passages. These results suggested that increasing the amount of time of bacteria in the intracellular environment did not improve the selection for hyper-replicating bacteria inside macrophages after three passages. Importantly, a high number of rough individual colonies (LPS O-antigen-negative mutants) was observed on agar plates after the three passages in murine macrophages.

The selection for mutants that lacked LPS O-antigen was further investigated. We passaged *S*. Typhimurium D23580 WT and 4/74 WT strains, in triplicates (each biological replicate was represented by a pool of 3 wells from 6-well plates), three times in macrophages. The conditions used were the same as described previously for the D23580 transposon library, collecting the intra-macrophage bacteria at 12 h p.i. and growing them in LB for 12 h between macrophage passages (S3C Fig). The number of rough and smooth colonies were counted after each passage (S3D and S3E Figs). Rough mutants identified by colony morphology on agar plates were validated by showing resistance to lysis by the Det7 [1] and 9NA [2] bacteriophages, two phages that bind to the *S*. Typhimurium LPS O-antigen receptor. The rough phenotype appeared after the second passage, with an average of 22% of colonies of D23580 WT, and 3% of 4/74 WT colonies. In the third passage, the proportion of rough mutants after 1.5 h of infection was 77% for D23580 WT, and 34% for 4/74 WT. After 12 h p.i. of the third passage, the averages were 48% in D23580 WT, and 17% in 4/74 WT. High numbers of rough mutants were recovered for each strain, D23580 WT and 4/74 WT, after growing the collected intra-macrophage bacteria of the third passage in LB, with an average of 95% in D23580 WT, and 68% in 4/74 WT. These results suggested stringent selection for LPS O-antigen-negative versions of the WT strains, consistent with a previous work [3]. Hölzer and colleagues reported that *S*. Typhimurium mutants lacking LPS O-

antigen are more invasive than the WT strains, being phagocytosed three times more by murine RAW264.7 macrophages [3].

This set of experiments led us to conclude that passaging the *S*. Typhimurium D23580 transposon library three times in murine RAW264.7 macrophages selected for mutants that lacked LPS O-antigen and exhibited decreased intra-macrophage replication.

## *S*. Typhimurium D23580 pBT1-cured mutant is slightly attenuated in murine macrophages

To determine whether the absence of the pBT1 plasmid in *S*. Typhimurium D23580 impacted upon virulence, the pBT1-cured mutant was used to infect murine RAW264.7 macrophages (S6A Fig). Proliferation of the intra-macrophage bacteria was measured by comparing bacterial counts at 15.5 h p.i. versus 1.5 h ($n$ = 3). The D23580 pBT1-cured mutant showed a small reduction of the fold-change replication inside macrophages compared with the WT strain. This result merits further investigation to determine whether pBT1 is required for optimal intra-macrophage replication.

## *S*. Typhimurium D23580 Δ*STM2475* mutant shows attenuation in murine macrophages, whereas D23580 *STM2475*$^{4/74SNP}$ does not

Canals and colleagues reported that the *STM2475* gene showed up-regulated expression, at the transcriptomic and proteomic levels, in D23580 compared with the *S*. Typhimurium ST19 strain 4/74 [4]. Closer inspection of the genetic region identified an indel difference between strains, an 'A' insertion in the −10 element of the *STM2475* promoter region, that was speculated to cause the differential expression (S6B Fig) [4]. The conservation of this indel was studied in a set of *S*. Typhimurium ST313 and ST19 genomes from Ashton and colleagues [5]. The indel was conserved in ST313 lineage 2 strains (S6C Fig). To study whether the overexpression of *STM2475* in D23580 could have an effect during infection of macrophages, mutants D23580 Δ*STM2475*::*frt* and D23580 *STM2475*$^{4/74SNP}$ were studied individually in our murine macrophage infection model (S6A Fig). The 4/74 Δ*STM2475*::*frt* and 4/74 *STM2475*$^{D23580SNP}$ mutants were included in the study for comparison. D23580 Δ*STM2475*::*frt* showed slight decrease in intra-macrophage replication compared with the WT strain. The D23580 *STM2475*$^{4/74SNP}$ mutant, carrying the promoter version of the 4/74 strain, did not show significant differences compared with the WT strain. In 4/74, neither the Δ*STM2475*::*frt* mutant nor the *STM2475*$^{D23580SNP}$ mutant showed significant differences compared with the WT strain. The reduction in the fold-change replication of the D23580 Δ*STM2475*::*frt* mutant warrants further investigation.

*S*. Typhimurium D23580 transposon mutants in the *STM2475* gene were not identified as attenuated in our TIS study in murine macrophages. These results could reflect differences in the two approaches, such as the use of a different post-infection time point (15.5 h in the individual proliferation assay versus 12 h in the TIS experiment), or potential trans-complementation in the TIS approach.

## The *STM1630* gene is expressed in *S*. Typhimurium 4/74, but not in D23580

The *STM1630* gene encodes a protein with unknown function in *S.* Typhimurium. The gene is located between two genes encoding effectors, *steB* and *sseJ*, and is absent in *S*. Typhi CT18 and Ty2 strains

[6]. Mutants in this gene are attenuated in calf and chicken infection models [7]. *STM1630* was identified as required for growth in our TIS study (S7A Fig), consistent with a previous *S.* Typhimurium TIS report [8]. To study whether the deletion of this gene had a fitness cost in D23580, the individual growth in LB of the D23580 Δ*STM1630*::*frt* mutant was compared to the growth of the WT strain (S7B Fig). No significant differences were observed between strains, concluding that this gene is not required for growth of *S*. Typhimurium D23580 in LB. This gene has been reported to contain an H-NS-binding site in *S*. Typhimurium SL1344 [9] (Fig 3C). These results suggest that the absence of transposons found in this gene in our TIS study could be due to H-NS-binding to the nucleotide sequence.

Consulting our previously published RNA-seq data, *STM1630* showed expression in 4/74 in an SPI-2-inducing medium (InSPI2), and inside murine macrophages [10,11]. In contrast, RNA-seq data in D23580 showed absence of expression of *STM1630* in the same *in vitro* growth conditions (S7C Fig) [4]. Furthermore, a comparison of the *STM1630* promoter region between *S*. Typhimurium D23580 and 4/74 indicated the presence of two consecutive SNPs, suggesting a disruption of the −10 element (S7D Fig). The conservation of the *STM1630* promoter SNPs was assessed in a set of 100 ST313 and ST19 genomes from Ashton and colleagues [11]. The two SNPs in D23580 were conserved in all ST313 strains tested, including lineages 1 and 2, and ST313 strains isolated from United Kingdom (S7E Fig) [5,12]. The differential expression of this gene between strains *S*. Typhimurium D23580 and 4/74 led us to investigate whether the absence of *STM1630* expression in D23580 in the intra-macrophage environment could have an effect in replication and survival inside macrophages. The D23580 WT and Δ*STM1630*::*frt* and *STM1630*$^{4/74\mathrm{SNP}}$ mutants were used to individually infect murine RAW264.7 macrophages (S7F Fig). For comparison, the 4/74 WT and the respective Δ*STM1630*::*frt* mutant were also studied. The fold-change replication in the intra-macrophage environment (15 h versus 1.5 h) did not show significant differences between the D23580 Δ*STM1630*::*frt* and *STM1630*$^{4/74\mathrm{SNP}}$ mutants compared with the D23580 WT strain. In contrast, the 4/74 Δ*STM1630*::*frt* mutant exhibited a significant increase of the fold-change value compared with the 4/74 WT strain. These results suggested a putative role for this gene inside murine macrophages in 4/74, but not in D23580.

# Supporting methods

## Construction of mutants in *S*. Typhimurium D23580 and 4/74 by λ Red recombineering

Mutants D23580 Δ*STM2475*::*frt*, 4/74 Δ*STM2475*::*frt*, D23580 Δ*STM1630*::*frt*, and 4/74 Δ*STM1630*::*frt* were constructed by λ Red recombineering following the described methodology in Materials and Methods. Generalized transduction using the bacteriophage P22 HT 105/1 *int-201* was performed for all constructions in D23580 and 4/74. Strains, plasmids and oligonucleotides used for obtaining these mutants are included in S10 and S11 Tables. For the *STM2475* mutants, oligonucleotides Fw-2475-P1 and Rv-2475-P2 were used for amplification of *aph* from the pKD4 plasmid. For mutants in the *STM1630* gene, PCR-amplification was performed using the Fw-STM1630-P1 and Rv-STM1630-P2 oligonucleotides.

## Construction of scarless single-nucleotide mutants in *S*. Typhimurium D23580 and 4/74

Single-nucleotide mutants were obtained using two different strategies that have been previously described [4,13]. The two-SNP substitution in D23580 *STM1630*$^{4/74SNP}$ was constructed using a single-stranded DNA recombineering strategy [14]. The STM1630p-474SNP oligonucleotide, carrying the two SNPs in the *STM1630* promoter region of 4/74, was transformed into D23580 containing the pSIM5-*tet* plasmid. D23580 recombinants were screened by PCR using a stringent annealing temperature with oligonucleotides Fw-STM1630p and Rv-STM1630p-474.

A second methodology based on the pEMG suicide plasmid was used for obtaining the D23580 *STM2475*$^{4/74SNP}$ and 4/74 *STM2475*$^{4/74SNP}$ mutants [15,16]. PCR-amplification was performed using oligonucleotides 2475-EcoRI-F and 2475-BamHI-R and D23580 or 4/74 as templates, depending on the indel difference to replace. PCR products were cloned into the pEMG plasmid using the BamHI and EcoRI restriction sites, and transformed into *E. coli* S17-1 λ*pir*. Recombinant plasmids were mobilized by conjugation into D23580 or 4/74, depending on the constructions carrying the D23580 or 4/74 indel version. For selection of transconjugants, M9 minimal medium supplemented with 0.2% glucose and Km was used for 4/74; and LB plates supplemented with Km and chloramphenicol 20 μg/mL were used for D23580. A second homologous recombination to remove the pEMG integrated plasmid was promoted by transformation of the pSW-2 plasmid, as previously described [15]. S10 and S11 Tables contain strains and plasmids, and the sequences of the oligonucleotides used for constructions.

## Supporting references


1. Casjens SR, Jacobs-Sera D, Hatfull GF, Hendrix RW. Genome Sequence of Salmonella enterica Phage Det7. Genome Announc. 2015;3. doi:10.1128/genomeA.00279-15

2. Casjens SR, Leavitt JC, Hatfull GF, Hendrix RW. Genome Sequence of Salmonella Phage 9NA. Genome Announc. 2014;2. doi:10.1128/genomeA.00531-14

3. Hölzer SU, Schlumberger MC, Jäckel D, Hensel M. Effect of the O-Antigen Length of Lipopolysaccharide on the Functions of Type III Secretion Systems in Salmonella enterica. Infect Immun. 2009;77: 5458–5470. doi:10.1128/IAI.00871-09

4. Canals R, Hammarlöf DL, Kröger C, Owen SV, Fong WY, Lacharme-Lora L, et al. Adding function to the genome of African Salmonella Typhimurium ST313 strain D23580. PLOS Biol. 2019;17: e3000059. doi:10.1371/journal.pbio.3000059

5. Ashton PM, Owen SV, Kaindama L, Rowe WPM, Lane CR, Larkin L, et al. Public health surveillance in the UK revolutionises our understanding of the invasive Salmonella Typhimurium epidemic in Africa. Genome Med. 2017;9. doi:10.1186/s13073-017-0480-7

6. Trombert AN, Berrocal L, Fuentes JA, Mora GC. S. Typhimurium sseJ gene decreases the S. Typhi cytotoxicity toward cultured epithelial cells. BMC Microbiol. 2010;10: 312. doi:10.1186/1471-2180-10-312

7. Morgan E, Campbell JD, Rowe SC, Bispham J, Stevens MP, Bowen AJ, et al. Identification of host-specific colonization factors of Salmonella enterica serovar Typhimurium. Mol Microbiol. 2004;54: 994–1010. doi:10.1111/j.1365-2958.2004.04323.x

8. Barquist L, Langridge GC, Turner DJ, Phan M-D, Turner AK, Bateman A, et al. A comparison of dense transposon insertion libraries in the Salmonella serovars Typhi and Typhimurium. Nucleic Acids Res. 2013;41: 4549–4564. doi:10.1093/nar/gkt148

9. Dillon SC, Cameron ADS, Hokamp K, Lucchini S, Hinton JCD, Dorman CJ. Genome-wide analysis of the H-NS and Sfh regulatory networks in Salmonella Typhimurium identifies a plasmid-encoded transcription silencing mechanism. Mol Microbiol. 2010;76: 1250–1265. doi:10.1111/j.1365-2958.2010.07173.x

10. Kröger C, Colgan A, Srikumar S, Händler K, Sivasankaran SK, Hammarlöf DL, et al. An Infection-Relevant Transcriptomic Compendium for Salmonella enterica Serovar Typhimurium. Cell Host Microbe. 2013;14: 683–695. doi:10.1016/j.chom.2013.11.010

11. Srikumar S, Kröger C, Hébrard M, Colgan A, Owen SV, Sivasankaran SK, et al. RNA-seq Brings New Insights to the Intra-Macrophage Transcriptome of Salmonella Typhimurium. PLOS Pathog. 2015;11: e1005262. doi:10.1371/journal.ppat.1005262

12. Okoro CK, Kingsley RA, Connor TR, Harris SR, Parry CM, Al-Mashhadani MN, et al. Intracontinental spread of human invasive Salmonella Typhimurium pathovariants in sub-Saharan Africa. Nat Genet. 2012;44: 1215–1221. doi:10.1038/ng.2423

13. Hammarlöf DL, Kröger C, Owen SV, Canals R, Lacharme-Lora L, Wenner N, et al. Role of a single noncoding nucleotide in the evolution of an epidemic African clade of Salmonella. Proc Natl Acad Sci U S A. 2018;115: E2614–E2623. doi:10.1073/pnas.1714718115

14. Sawitzke JA, Costantino N, Li X-T, Thomason LC, Bubunenko M, Court C, et al. Probing cellular processes with oligo-mediated recombination and using the knowledge gained to optimize recombineering. J Mol Biol. 2011;407: 45–59. doi:10.1016/j.jmb.2011.01.030

15. Owen SV, Wenner N, Canals R, Makumi A, Hammarlöf DL, Gordon MA, et al. Characterization of the Prophage Repertoire of African Salmonella Typhimurium ST313 Reveals High Levels of

Spontaneous Induction of Novel Phage BTP1. Front Microbiol. 2017;8. doi:10.3389/fmicb.2017.00235

16. Martínez-García E, Lorenzo V de. Engineering multiple genomic deletions in Gram-negative bacteria: analysis of the multi-resistant antibiotic profile of Pseudomonas putida KT2440. Environ Microbiol. 2011;13: 2702–2716. doi:10.1111/j.1462-2920.2011.02538.x
