# Supplementary figures and images for: The fitness landscape of the African Salmonella Typhimurium ST313 strain D23580 reveals unique properties of the pBT1 plasmid
Source: PLoS Pathog. 2019 Sep 27;15(9):e1007948. doi: 10.1371/journal.ppat.1007948 (PMC6785131; doi:10.1371/journal.ppat.1007948)

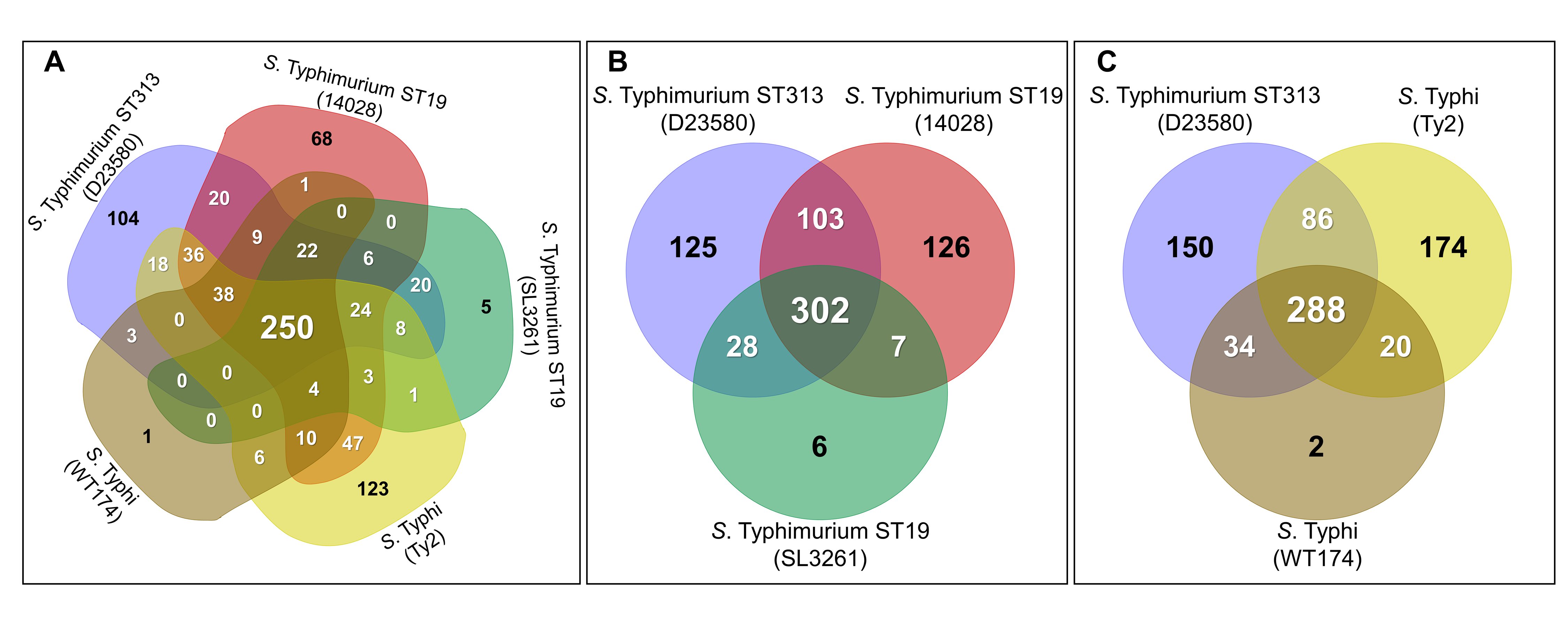

Supplement: S1 Fig — (A) Comparative analysis of S. Typhimurium D23580 required genes with previously identified required genes in TIS studies in S. Typhimurium [21,25], and S. Typhi [20,21]. SL3261 was derived from SL1344; and WT174 was derived from Ty2. For the previously published studies, only genes that shared an ortholog in D23580 were included for the analysis. Individual Venn diagram analyses including a comparison with only S. Typhimurium (B) and S. Typhi (C) strains were also generated. (TIF) [file ppat.1007948.s002.tif]

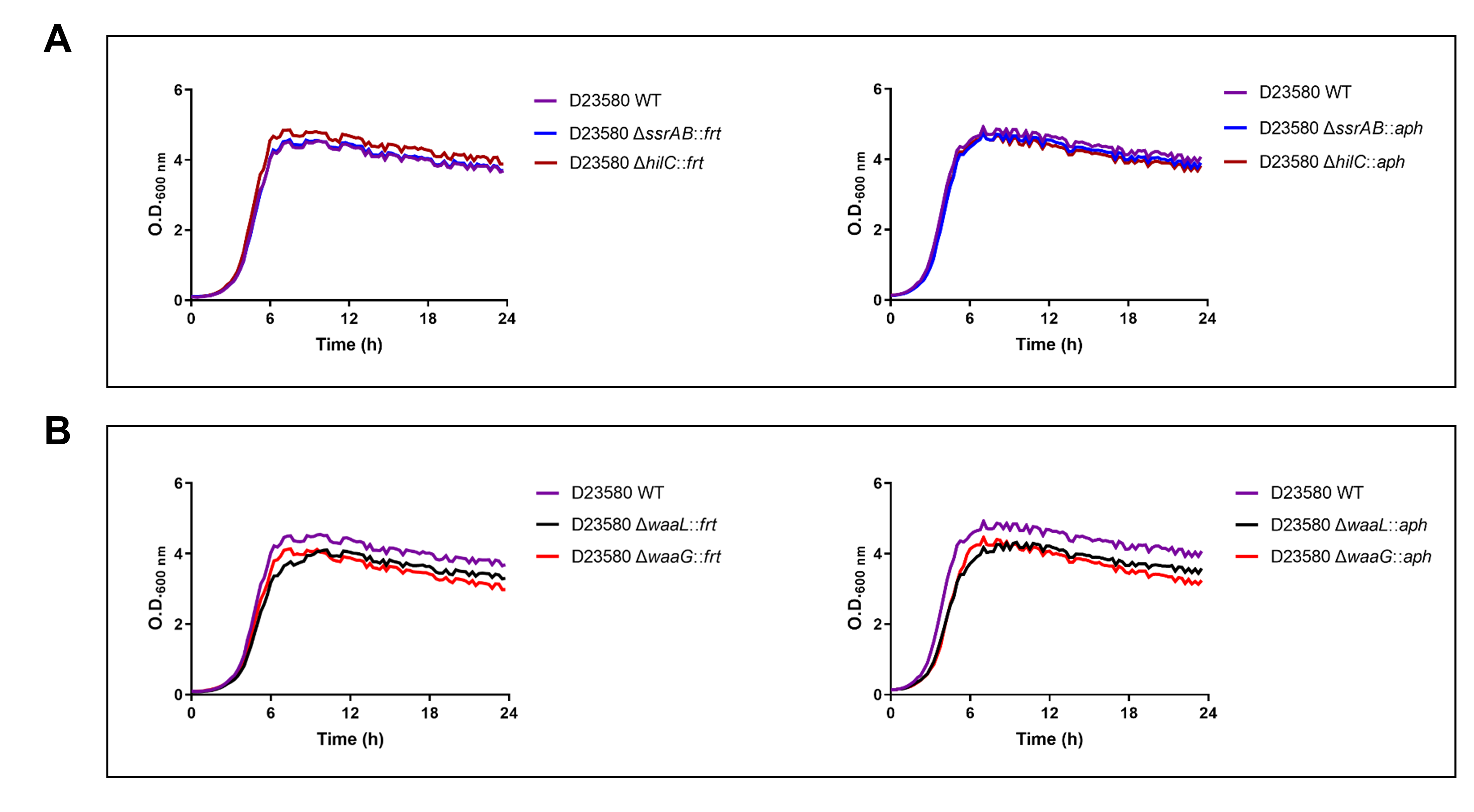

Supplement: S2 Fig — Individual growth curves, in LB medium, of the Km resistant versions (aph, n = 8) and the deletion versions (frt, n = 7) of (A) a SPI-1 mutant (hilC), a SPI-2 mutant (ssrAB), and (B) LPS mutants (waaL and waaG). (TIF) [file ppat.1007948.s003.tif]

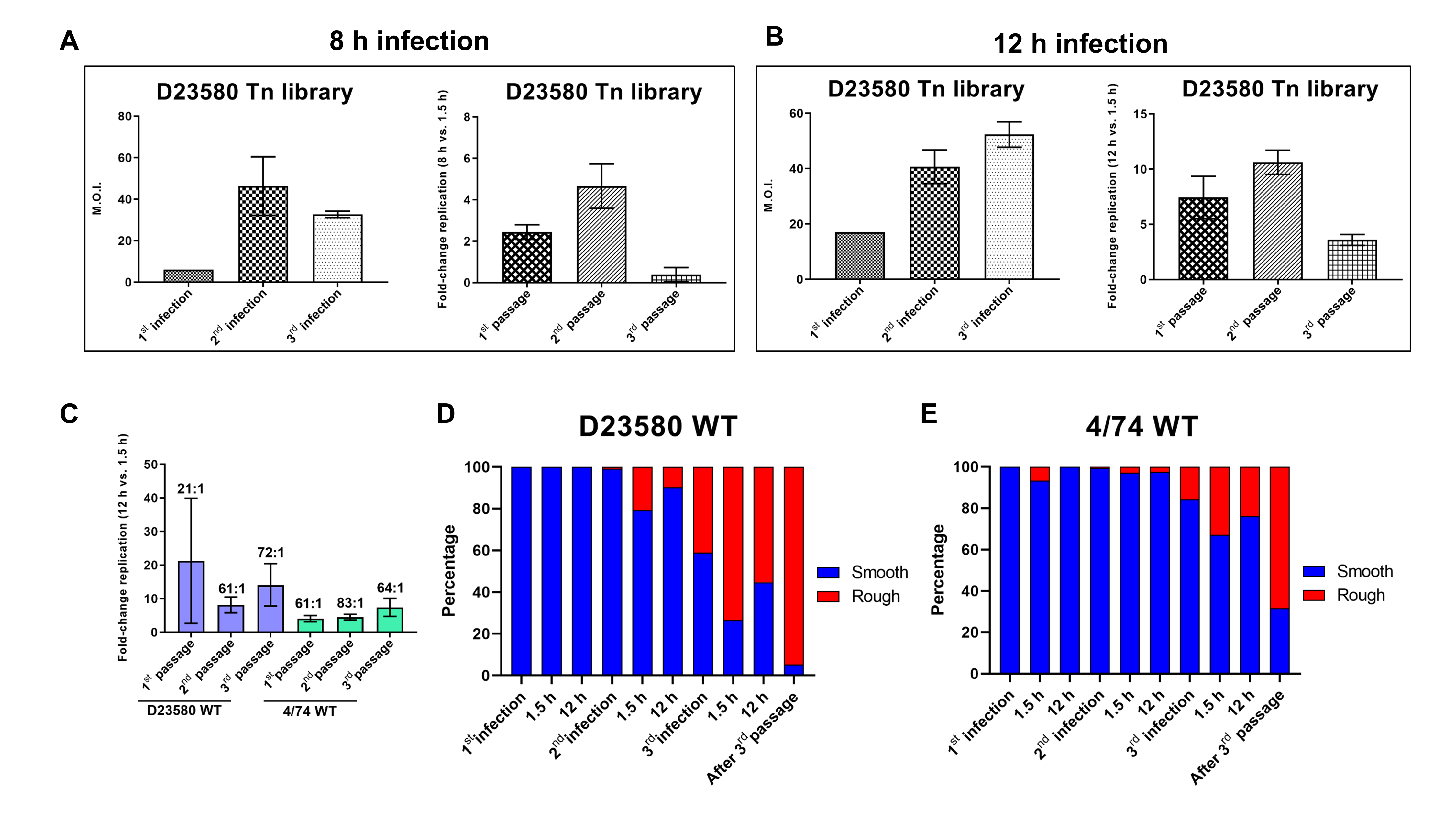

Supplement: S3 Fig — (A) M.O.I. (number of bacterial cells used to infect one macrophage) of the D23580 transposon library infecting murine RAW264.7 macrophages for 8 h, used in the first (n = 1), second (n = 3) and third infections (n = 3). Fold-change replication of the intra-macrophage bacteria (8 h versus 1.5 h) of the D23580 transposon library seen after each passage. Error bars show standard deviation (n = 3). (B) M.O.I. and fold-change replication of the intra-macrophage bacteria of the D23580 transposon library at 12 h p.i. (C) Fold-change replication of the D23580 WT and 4/74 WT strains inside murine RAW264.7 macrophages. M.O.I.s calculated for one of the three biological replicates are indicated at the top of each bar (n = 3). (D) The percentage of rough mutants increased after passages of S. Typhimurium D23580 WT in macrophages and LB (first and second infections, n = 1; third infection, n = 3; after third passage, n = 3); (E) and in S. Typhimurium 4/74 WT (first and second infections, n = 1; third infection, n = 2; after third passage, n = 3). (TIF) [file ppat.1007948.s004.tif]

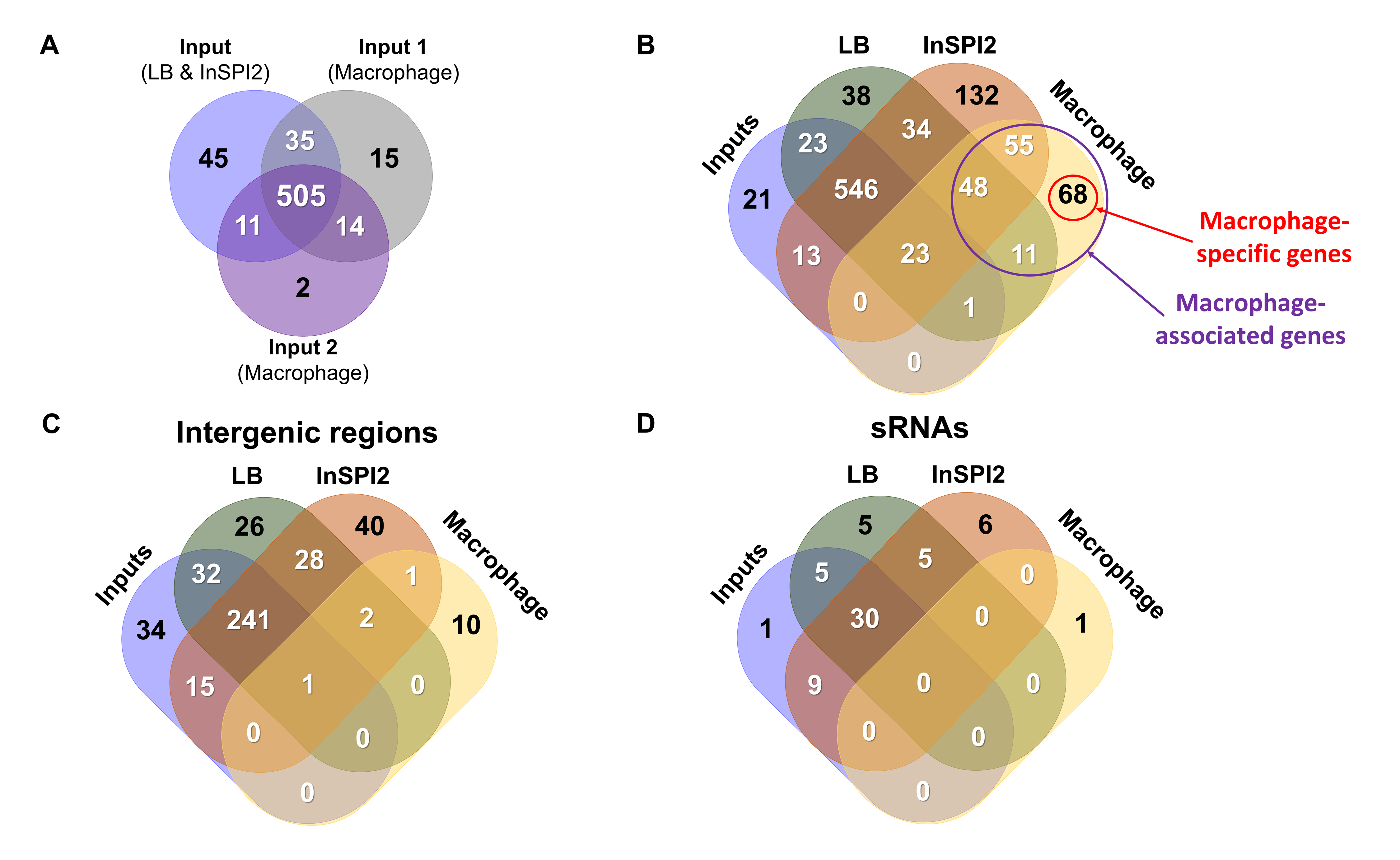

Supplement: S4 Fig — (A) 10% of the S. Typhimurium D23580 genes are required for growth in all three LB input mutant pools. (B) Identification of S. Typhimurium D23580 “macrophage-specific” and “macrophage-associated” genes. The Venn diagram compares the 206 S. Typhimurium D23580 genes that showed attenuation in RAW264.7 macrophages when disrupted by a transposon insertion with required genes in the three inputs (Inputs), and the LB and InSPI2 outputs. (C) Venn diagrams including only intergenic regions, and (D) sRNAs. (TIF) [file ppat.1007948.s005.tif]

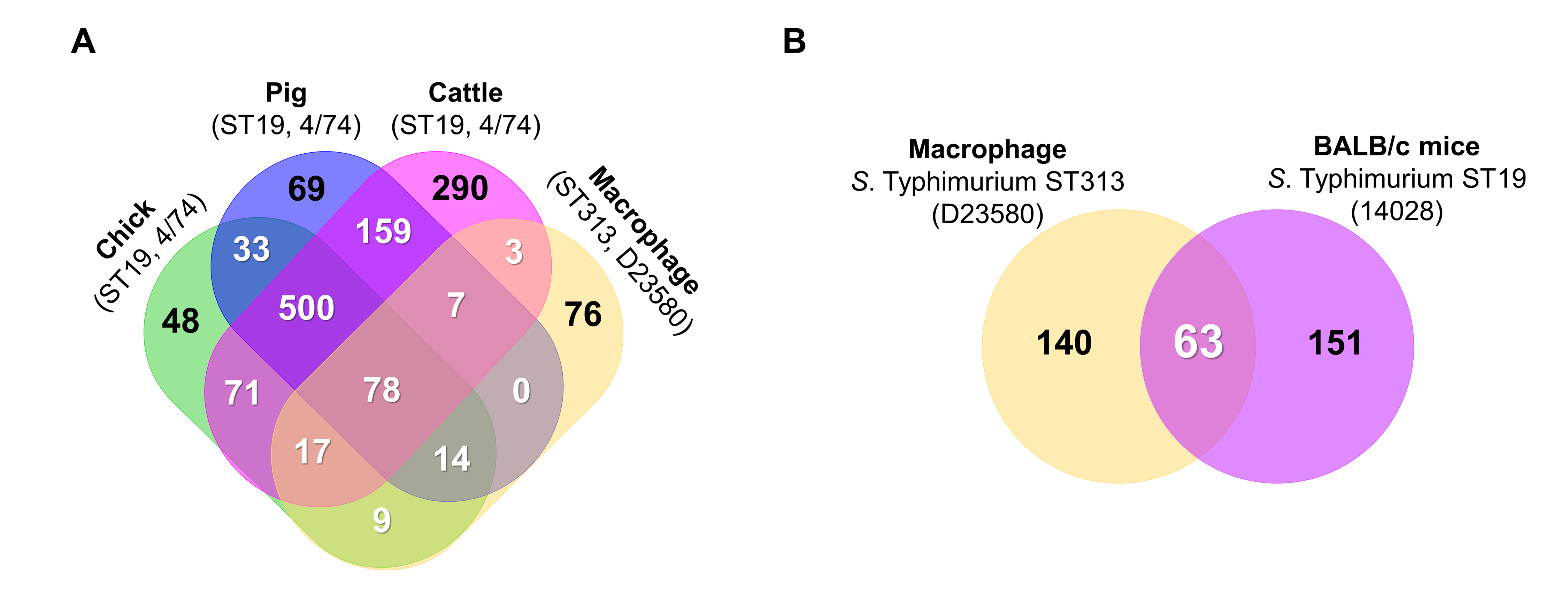

Supplement: S5 Fig — (A) 63% of the D23580 macrophage-attenuated genes are important for virulence of S. Typhimurium 4/74 in food-related animal infection models [51]. Only orthologous chromosomal and pSLT plasmid genes were included for the analysis. (B) D23580 macrophage-attenuated genes compared to S. Typhimurium 14028 genes associated to virulence in BALB/c mice [52]. Only orthologous chromosomal genes were included for the analysis. (TIF) [file ppat.1007948.s006.tif]

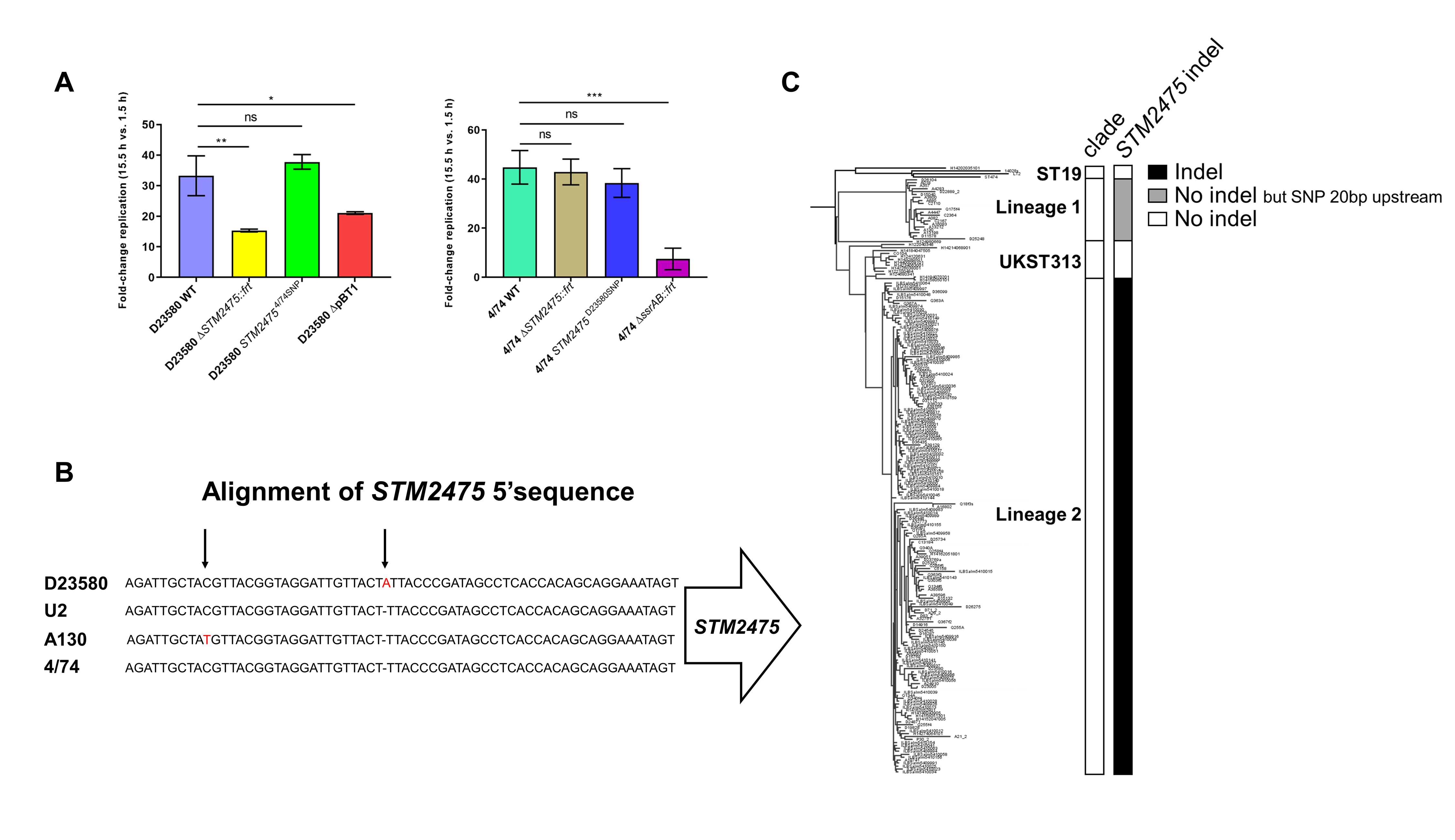

Supplement: S6 Fig — (A) Intra-macrophage proliferation assays of the D23580 WT, D23580 ΔSTM2475::frt, D23580 STM24754/74SNP, D23580 ΔpBT1; and 4/74 WT, 4/74 ΔSTM2475::frt, 4/74 STM2475D23580SNP, 4/74 ΔssrAB::frt. Bars represent average of three independent biological replicates and standard deviation. Significant differences indicate P-value: ***, 0.0002; **, 0.0011; *, 0.0116; ns, not significant. (B) Alignment of the STM2475 promoter region in four S. Typhimurium strains. (C) Conservation of the nucleotide indel in S. Typhimurium ST313 strains. (TIF) [file ppat.1007948.s007.tif]

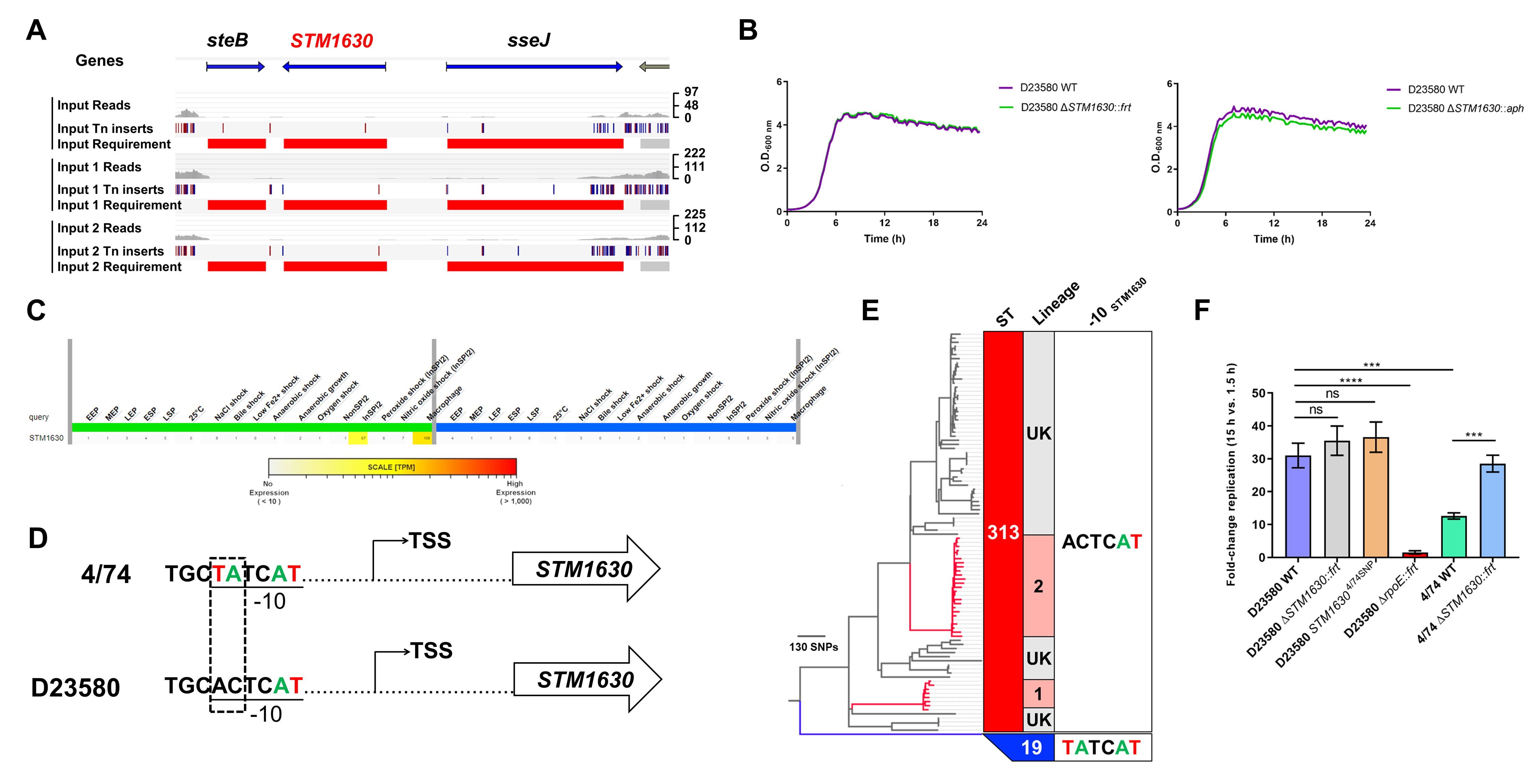

Supplement: S7 Fig — (A) Transposon insertion profile of the STM1630 region from our D23580 Dalliance genome browser. (B) There were not significant differences in growth in LB between D23580 WT and the ΔSTM1630 mutants, with or without the Km resistance cassette. (C) Absolute expression levels of STM1630 in S. Typhimurium D23580 and 4/74 (extracted from Canals and colleagues [15]). Values represent TPM, TPM ≤10 means no expression. (D) Disruption of STM1630 −10 box in the promoter region: two SNP-difference between 4/74 and D23580. (E) The D23580 isoform is conserved in all ST313 genomes analyzed, including lineage 1 and 2 and UK-ST313 strains described in Ashton and colleagues [87]. BLASTn was used to identify the genotype of the STM1630 transcriptional start site −10 region in all genomes and the results were visualized in the context of the phylogenetic tree from Ashton and colleagues [87]. (F) Intra-macrophage proliferation assays of the D23580 and 4/74 WT strains, the ΔSTM1630::frt mutants for D23580 and 4/74, and the D23580 STM16304/74SNP mutant. Bars represent average of three independent biological replicates and standard deviation. Significant differences indicate P-value: ****, <0.0001; ***, <0.001; ns, not significant. (TIF) [file ppat.1007948.s008.tif]
