# Supplementary material for: The fitness landscape of the African Salmonella Typhimurium ST313 strain D23580 reveals unique properties of the pBT1 plasmid
Source: PLoS Pathog. 2019 Sep 27;15(9):e1007948. doi: 10.1371/journal.ppat.1007948 (PMC6785131; doi:10.1371/journal.ppat.1007948)
Supplement: S5 Table — (PDF) [file ppat.1007948.s013.pdf]

Strains	Lag time (h)	Maximum OD_{600}	Maximal growth rate (doubling / h)
D23580 WT	3.2	4.55	1.03
D23580 Δ*ssrAB*::*frt*	3.3	4.58	1.03
D23580 Δ*hilC*::*frt*	3.2	4.86	0.99
D23580 Δ*waaL*::*frt*	3.4	4.11	0.97
D23580 Δ*waaG*::*frt*	3.4	4.14	1.00
D23580 Δ*STM1630*::*frt*	3.2	4.57	1.06

Strains	Lag time (h)	Maximum OD_{600}	Maximal growth rate (doubling / h)
D23580 WT	2.7	4.92	0.98
D23580 Δ*ssrAB*::*aph*	2.9	4.73	0.95
D23580 Δ*hilC*::*aph*	2.9	4.76	0.94
D23580 Δ*waaL*::*aph*	2.9	4.32	1.1
D23580 Δ*waaG*::*aph*	3.2	4.46	0.99
D23580 Δ*STM1630*::*aph*	2.8	4.61	0.96
D23580 ΔpBT1	2.9	5.09	1.01

Strains	Lag time (h)	Maximum OD_{600}	Maximal growth rate (doubling / h)
D23580 WT	2.7	4.61	0.92
D23580 Δ$cysS^{pBT1}$::*aph*	4.2	4.69	1.37
D23580 Δ$cysS^{pBT1}$::*frt*	4.4	4.72	1.35
