# Supplementary material for: The fitness landscape of the African Salmonella Typhimurium ST313 strain D23580 reveals unique properties of the pBT1 plasmid
Source: PLoS Pathog. 2019 Sep 27;15(9):e1007948. doi: 10.1371/journal.ppat.1007948 (PMC6785131; doi:10.1371/journal.ppat.1007948)
Supplement: S10 Table — (PDF) [file ppat.1007948.s018.pdf]

Bacterial Strains	Source	Identifier
S. Typhimurium D23580 (ST313)	[1,2]	JH3621
S. Typhimurium 4/74 (ST19)	[3]	JH3676
S. Typhimurium D23580 Δ*cysS*^{pBT1}::*aph*	[2]	JH4298
S. Typhimurium D23580 Δ*cysS*^{pBT1}::*frt*	[2]	JH4299
S. Typhimurium D23580 ΔpBT1	[2]	JH4300
S. Typhimurium D23580 Δ*ssrAB*::*aph*	[4]	SO-46
S. Typhimurium D23580 Δ*ssrAB*::*frt*	[4]	SO-53
S. Typhimurium D23580 Δ*hilC*::*aph*	[4]	JH4327
S. Typhimurium D23580 Δ*hilC*::*frt*	[4]	JH4328
S. Typhimurium D23580 Δ*waaG*::*aph*	[5]	JH3917
S. Typhimurium D23580 Δ*waaG*::*frt*	[4]	JH4187
S. Typhimurium D23580 Δ*waaL*::*aph*	[4]	SO-13
S. Typhimurium D23580 Δ*waaL::frt*	[4]	SO-51
S. Typhimurium D23580 Δ*argA*::*frt*	This study	JH4372
S. Typhimurium 4/74 Δ*argA*::*frt*	This study	JH4369
S. Typhimurium D23580 Nal^{R}	This study	JH3796
S. Typhimurium D23580 Δ*STM2475*::*frt*	This study	JH4357
S. Typhimurium D23580 *STM2475*$^{4/74SNP}$	This study	JH4361
S. Typhimurium 4/74 Δ*STM2475*::*frt*	This study	JH4359
S. Typhimurium 4/74 *STM2475*^{D23580SNP}	This study	JH4360
S. Typhimurium D23580 Δ*STM1630*::*aph*	This study	JH4363
S. Typhimurium D23580 Δ*STM1630::frt*	This study	JH4366
S. Typhimurium D23580 *STM1630*$^{4/74SNP}$	This study	JH4362
S. Typhimurium D23580 Δ*rpoE::frt*	[4]	JH4235
S. Typhimurium 4/74 Δ*STM1630::frt*	This study	JH4365
**Plasmids**		
frt-aph-frt cassette template plasmid; Km^{R}	[6]	pKD4
λ Red recombination plasmid, temperature-inducible; Tc^{R}	[7]	pSIM5-*tet*
FLP recombinase expression plasmid; Tc^{R}	[8]	pCP20-TcR
pET28a expression vector; His-tag, Km^{R}	Novagen	pET28a
pET28a carrying *cysS*^{chr}	This study	pET28a-*cysS*^{chr}
pET28a carrying *cysS*^{pBT1}	This study	pET28a-*cysS*^{pBT1}

Supporting references

1. Kingsley RA, Msefula CL, Thomson NR, Kariuki S, Holt KE, Gordon MA, et al. Epidemic multiple drug resistant Salmonella Typhimurium causing invasive disease in sub-Saharan Africa have a distinct genotype. Genome Res. 2009;19: 2279–2287. doi:10.1101/gr.091017.109

2. Canals R, Hammarlöf DL, Kröger C, Owen SV, Fong WY, Lacharme-Lora L, et al. Adding function to the genome of African Salmonella Typhimurium ST313 strain D23580. PLOS Biol. 2019;17: e3000059. doi:10.1371/journal.pbio.3000059

3. Rankin JD, Taylor RJ. The estimation of doses of Salmonella typhimurium suitable for the experimental production of disease in calves. Vet Rec. 1966;78: 706–707.

4. Lacharme-Lora L, Owen SV, Blundell R, Canals R, Wenner N, Perez-Sepulveda B, et al. The use of chicken and insect infection models to assess the virulence of African Salmonella Typhimurium ST313. PLoS Negl Trop Dis. 2019;13: e0007540. doi:10.1371/journal.pntd.0007540

5. Hammarlöf DL, Kröger C, Owen SV, Canals R, Lacharme-Lora L, Wenner N, et al. Role of a single noncoding nucleotide in the evolution of an epidemic African clade of Salmonella. Proc Natl Acad Sci U S A. 2018;115: E2614–E2623. doi:10.1073/pnas.1714718115

6. Datsenko KA, Wanner BL. One-step inactivation of chromosomal genes in Escherichia coli K-12 using PCR products. Proc Natl Acad Sci U S A. 2000;97: 6640–6645.

7. Koskiniemi S, Pränting M, Gullberg E, Näsvall J, Andersson DI. Activation of cryptic aminoglycoside resistance in Salmonella enterica. Mol Microbiol. 2011;80: 1464–1478. doi:10.1111/j.1365-2958.2011.07657.x

8. Kintz E, Davies MR, Hammarlöf DL, Canals R, Hinton JCD, van der Woude MW. A BTP1 prophage gene present in invasive non-typhoidal Salmonella determines composition and length of the O-antigen of the lipopolysaccharide. Mol Microbiol. 2015;96: 263–275. doi:10.1111/mmi.12933
