# Supplementary material for: The fitness landscape of the African Salmonella Typhimurium ST313 strain D23580 reveals unique properties of the pBT1 plasmid
Source: PLoS Pathog. 2019 Sep 27;15(9):e1007948. doi: 10.1371/journal.ppat.1007948 (PMC6785131; doi:10.1371/journal.ppat.1007948)
Supplement: S11 Table — (PDF) [file ppat.1007948.s019.pdf]

Oligonucleotide	Oligonucleotide sequence (5'→3')
PE PCR Tn-1	AATGATACGGCGACCACCGAGATCTACACTCTTTCCCTACACGACGCTCTTCCGATCT**CGTGAT**GCTTCAGGGTTGAGATGTGTA
PE PCR Tn-4	AATGATACGGCGACCACCGAGATCTACACTCTTTCCCTACACGACGCTCTTCCGATCT**TGGTCA**GCTTCAGGGTTGAGATGTGTA
PE PCR Tn-5	AATGATACGGCGACCACCGAGATCTACACTCTTTCCCTACACGACGCTCTTCCGATCT**CACTGT**GCTTCAGGGTTGAGATGTGTA
PE PCR Tn-7	AATGATACGGCGACCACCGAGATCTACACTCTTTCCCTACACGACGCTCTTCCGATCT**GATCTG**GCTTCAGGGTTGAGATGTGTA
PE PCR Tn-10	AATGATACGGCGACCACCGAGATCTACACTCTTTCCCTACACGACGCTCTTCCGATCT**AAGCTA**GCTTCAGGGTTGAGATGTGTA
PE PCR Tn-12	AATGATACGGCGACCACCGAGATCTACACTCTTTCCCTACACGACGCTCTTCCGATCT**TACAAG**GCTTCAGGGTTGAGATGTGTA
Fw-argA-P1	TCGAATAATAATTCACTAGTGGGGCGCGCAATGATAAAGGAACGTGTGTAGGCTGGAGCTGCTTC
Rv-argA-P2	CCTGATAAGCGCAGCGCCATCAGGCCCCCCTTGTTATCCTAAATCCATATGAATATCCTCCTTAG
cysS pBT1 pET28a OH Fwd	GCCGCGCGGCAGCCATATGCTAAAGATATACAATACATACACTCGC
cysS pBT1 pET28a OH Rev	TGGTGGTGGTGGTGCTTATTTACGGCGCCAGGTTGT
D23580 chromosomal cysS pET28a OH Fwd	GCCGCGCGGCAGCCATATGTTAAAAATTTTTAATACACTGACACGCC
D23580 chromosomal cysS pET28a OH Rev	TGGTGGTGGTGGTGCTTACTTACGTCGCCAGGTGG
2475-EcoRI-F	GGAATTCCGTACCGCTGAGTGAGGAA
2475-BamHI-R	CGGGATCCGGCCTAAAAGGCGCAATTA
Fw-2475-P1	ACAGCAGGAAATAGTATGAAAATAAATAATGGCCCCGTATTATGCGTGTAGGCTGGAGCTGCTTC
Rv-2475-P2	ACCTGTTCTGTAGGCTGTGCAAAATTACTTCGTAGACTCAGGCGCCATATGAATATCCTCCTTAG
STM1630p-474SNP	TCTTTGCCTTAGCCAGGCGCACACTCAATAATGATAGCAGTCAGATAATATGTACCAGGCATTAACCTCA
Fw-STM1630p	ACAAAACCGCTGCCAAAA
Rv-STM1630p-474	GGTACATATTATCTGACTGCTA
Fw-STM1630-P1	AACTCTTCGATGCTTTTGGCCTATTTAGGTGGTTTGGGATGGGGAGTGTAGGCTGGAGCTGCTTC
Rv-STM1630-P2	AAGTTCTGCCGTCATTTCACGCTCTTGCCAACCAGCATTTTATTACATATGAATATCCTCCTTAG
